# 

**Published:** 1870-07

**Authors:** 


### Books and Pamphlets Received.

Annual Report of the Metropolitan Board of Health.—Medical opinion of Chas. B Lee, M.D.—Constitution of the Toronto Eye and Ear Infirmary.—Annual Catalogue of Bellevue Hospital Medical College, N. Y.—Transactions of the New York Academy of Medicine.—Circular, University of Albany.—Circular, N. Y. State Inebriate Asylum.—Prychopathic Hospltal of the Future, by Pliny Earl.—Address: Berkshire Medical Institute, by Pliny Earle.—Prospective Provision for the Insane, by Pliny Earle.—State Lunatic Hospital Report, at Northampton.—The Bromhides, by Z. C. Mc Elroy.—New York Ophthalmic and Au[illegible]al Institutes Report.—Medical Department.—Uuiversity of Pennsylvania, Catalogue and Announcement—Medical Legal Study, by Wm. A. Hammond.—Woman's Medical College.—New York Infirmary, Catalogue.—Treatment of Croup, by Fordyce Barker.

PRIZE ESSAYS.—At the Annual Meeting of the Medical Society of the State of New York, held in Albany, February, 1870, a prize of one hundred dollars was offered for the best essay on any medical subject. Also a prize of one hundred dollars was offered by Dr. Hiram Corliss, for the best essay on the cause, prevention and cure of Tubercular Phthisis. Both essays must be sent to one of the Committee on Prize Essays by the first of December next, and be subject to the regulations of the society regarding Prize Essays, as found on page 71 of the transactions for 1869. The Committee are Drs. Thomas F. Rochester, of Buffalo, Sandford Eastman, of Buffalo and Henry W. Dean of Rochester.

W. H. BAILEY, SECRETARY.

